# Price prediction of polyester yarn based on multiple linear regression model

**DOI:** 10.1371/journal.pone.0310355

**Published:** 2024-09-12

**Authors:** Wenyi Qiu, Qingjun Mao, Chen Liu

**Affiliations:** 1 School of Global Education & Development, University of Chinese Academy of Social Sciences-University of Stirling, Beijing, China; 2 Department of Mechanical Engineering, Tsinghua University, Beijing, China; 3 Industrial Development Center, Zhejiang Materials Industry Yuantong Automobile Group Co., Ltd., Hangzhou, China; Gdańsk University of Technology: Politechnika Gdanska, POLAND

## Abstract

China’s polyester textile industry is one of the notable contributors to national economy. This paper takes polyester yarn, core raw material in polyester textile industry chain, as research object, and deeply explores its price indicators and risk hedging mechanisms through multiple linear regression models and Holt-Winters approaches. It is worth mentioning that with continuous development of digital technology, digital transformation of production lines and warehouses has become an important development feature in various industries. This study also actively complies with this trend, and innovatively incorporates the upstream and downstream production line start-up rates into price prediction model. Through this initiative, we can more comprehensively consider the impact of supply and demand changes on price of polyester yarn, thus making prediction results more closely reflect the actual market situation. This quantitative analysis method undoubtedly provides new ideas for enterprises to better grasp market dynamics in digital era.

## 1. Introduction

Warp knitting is an important weaving process, which refers to the knitting of warp wale into fabrics. Its upstream industry is polyester chemical fiber, and its downstream industries include clothing, home textiles, etc. Before the 1970s, the warp knitting industry was mainly located in Europe and the United States. Chen pointed out in his research that the great development of China’s warp knitting industry began in the 1970s, benefiting from the development of China’s chemical fiber industry [1]. Presently, China is the largest base for warp knitting industry in the world, and the market share is still increasing. At the same time, the regional integration features are obvious. Ge et al. summarized that more than 90% of the enterprises in the Zhejiang Haining Warp Knitting Industrial Park were engaged in warp knitting industry, with output value accounting for more than 90% of the total output value of the whole district [2].

With the increasing number of market participants, the role of market mechanisms has become increasingly prominent. We have observed an increasing sensitivity among participants in the industry chain towards price movements. Bruce et al. pointed out that the supply chain in the textiles industry was complex. The supply chain is relatively long, with a number of parties involved. Consequently, careful management of the supply chain is required in order to reduce lead times and achieve quick response [3]. Changes in supply and demand will directly or indirectly affect price trends, resulting in complicating price changes. Dai analyzed the main factors affecting the operational performance of the polyester industry chain from the perspectives of value chain, supply chain, enterprise partnership, spatial agglomeration mode, and proposed that risk management is an important tool for enterprise operation [4].

For example, from June 10, 2022 to July 15, 2022, the price of PTA, the main raw material of polyester yarn, fell from 7,562 yuan/ton to 5,280 yuan/ton in less than a month. Prices have fallen by nearly 30%. Correspondingly, the price of mainstream specification polyester yarn has fallen from 6,015 yuan/ton to 5,080 yuan/ton. The price of a single ton has fallen by nearly 1,000 yuan. The plummeting prices of PTA and polyester yarn have had a huge impact on the stability of the supply chain.

Facing the violent fluctuation of raw material prices, the traditional manufacturing industry lacks sufficient risk management capabilities. Fischl et al. mentioned that risks related to the purchase prices of industrial consumption factors (raw materials, semi-finished/finished goods, auxiliary materials, and operating materials) exerted an increasing influence on manufacturing companies’ business continuity and economic sustainability [5]. During the period of sharp price declines, when companies purchased raw materials, the price of polyester filament was at a high point. But the price dropped when they sold their products, and the profit of the company was compressed. Some companies even experienced the inversion of the sales price and the cost price.

The price of polyester yarn is affected by the macroeconomic environment and its supply and demand. Chen et al. pointed out that the operation demand of the textile industry supply chain came from various information supports. The quality of information such as the market demand and price prediction of final products, the yield and price prediction of raw materials affects the effective operation of the supply chain [6]. Das and Chakrabarti proposed a Multilayer Perceptron (MLP) approach, developed efficient forecasting models using it for the Wholesale Price Index (WPI) of all the twenty-five individual items of the manufacture of the textiles group of India [7]. Lorente-Leyva et al. focused on the demand forecasting for textile products by comparing a set of classic methods such as ARIMA, STL Decomposition, Holt-Winters and machine learning, Artificial Neural Networks, Bayesian Networks, Random Forest, Support Vector Machine [8].

However, most price forecasting studies only consider the historical prices of related products. Due to the lack of data sources for key data such as industry start-up rates, it is difficult to quantitatively incorporate changes in supply and demand into analytical models. Yıldız and Møller stated that the complexity of manufacturing systems, on-going production and existing constraints on the shop floor remained among the main challenges for the analysis, design and development of the models in product, process and factory domains [9]. With the development of the industry, more and more companies are beginning to carry out digital construction to support complex manufacturing systems and continuous production. We have observed that in the industrial clusters, some leading companies with years of in-depth understanding and knowledge of the industry have begun to actively explore and innovate, with a particular focus on digitalization and the construction of virtual factories. According to Li’s research, the implementation of enterprise digitalization and the construction of industrial internet platforms can achieve rapid interaction of industrial data, promoting the integrated development of industry chains, value chains, innovation chains, and capital chains [10]. Up to now, a large amount of production and operation data from the warp knitting textile industry chain has been connected to the cloud, providing support for studying price influencing factors.

Therefore, this paper innovatively considers the capacity utilization rates of upstream and downstream industries in the price forecasting model, quantitatively incorporating changes in supply and demand into the analytical model. Leveraging the data accumulated through industrial digitization and integrating it with the public data from China’s Commodity Exchanges, it has established a solid foundation for studying the price transmission mechanism of polyester yarn and identifying its key price indicators. This holds significant importance for comprehensively grasping market price fluctuations and stabilizing the supply chain.

## 2. Literature review

Recent literature provides various perspectives on dynamic analysis of commodity price distribution and its correlated factors. Zhang et al. utilized bibliometrics to trace the development of research on commodity prices, and conducted statistical and co-citation analyses. It was found that the research hotspots in this field are concentrated on four aspects: factors influencing commodity prices, the impact of price fluctuations on the macroeconomy, forecasts of commodity prices, and the financialization of commodities [11]. Li and Chavas investigated the role of futures markets and their dynamic effects on the stability of commodity prices based on a quantile vector autoregression (QVAR) model of the marginal distributions of futures and spot prices, and a copula of their joint distribution. The paper finds evidence of nonlinear price dynamics that depend on the maturity of the futures contract and documents how marginal price distributions and associated moments evolve over time [12]. Le et al. examined the dynamic effect of oil prices on other energy prices based on asymmetric cointegration and dynamic multipliers in a nonlinear ARDL framework. The paper identifies positive relationships between oil price and the prices of other energy commodities [13]. Landajo and Presno addressed the problem of testing for persistence in the effects of the shocks affecting the prices of renewable commodities based on stationarity testing conditional on the number of changes detected and the detection of change points, and finds non-linear features that often coincide with well-known political and economic episodes [14].

Pani et al. examined the price discovery function of the bullion, metal, and energy commodity futures and spot prices through the Granger causality and Johansen–Juselius cointegration tests. The findings of the study suggest the market participants for implementing hedging and arbitrage strategies [15]. Ubilava conducted a comparison of multistep commodity price forecasts using direct and iterated smooth transition autoregressive methods (STAR), and finds that the STAR models are in most instances inferior to the basic autoregressive framework for multistep commodity price forecasting [16]. Chatnani analyzed the long hedge strategy using the Multi Commodity Exchange (MCX) of India listed lead contracts to identify the advantages and disadvantages of hedging with futures contracts, and examine how hedging replaces price risk with basis risk [17]. Koziol and Treuter analyzed the impact of speculative trading in agricultural commodity markets on major economic quantities. It identifies crucial variables determining whether speculative trading is beneficial or dangerous, including the correlation between the speculators’ portfolio and the commodity prices, the risk premium of the forward, and the producer’s gains [18].

The abovementioned literature review provides pivotal information on the methodology of commodity price forecast and impact of related hedging and speculation activities. The polyester textile industry chain is very long, so there are many factors affecting its price. For instance, macro factors such as world macroeconomic changes, exchange rate changes, and unexpected political events, as well as government macro-control, industrial policy, tariff adjustment, chemical fiber industry cycle, business operating costs, crude oil price fluctuation, market demand, trade disputes and other micro factors. Therefore, it is precisely because of a great number of influencing factors and huge price volatility that a lot of financial institutions participate in the trading of PTA and MEG futures contracts and conduct speculative operations.

Thus, it is of great significance to find the factors of significant correlation and identify the price transmission mechanism. In this way, it is achievable to grasp the market price trend and guide the entity enterprises to effectively hedge the risk of price fluctuations.

## 3. Method

Multiple linear regression model has significant statistical significance, and is widely used in management disciplines and economics. Multiple regression analysis refers to the use of regression equations to quantitatively explain the linear dependence between dependent variables and two or more independent variables. It is used to find the mathematical expression that best represents the relationship between independent variables and dependent variables [19–21]. The analysis process of multiple regression analysis generally includes correlation analysis, significance analysis, regression detection, etc.

Let the dependent variable be *Y*, and the k independent variables are *X*_1_, *X*_2_……*X*_k_. The general form of the multiple linear regression model is:

$$Y=\beta_{0}+\beta_{1}X_{1}+\beta_{2}X_{2}+\cdots +\beta_{k}X_{k}+\varepsilon$$ (1)

*β*_1_ is regression constant, *β*_1_, *β*_2_, …, *β_k_* are regression coefficients, and *ε* is random error term.

The purpose of this paper is to investigate the key factors affecting the price trend of polyester yarn, and to build a multiple linear regression model to predict the future price trend.

Thus, this paper selected the daily average price of one mainstream specification of polyester yarn, 50D/24F FDY (Fully Drawn Yarn), as the dependent variable. The data is generated from data services purchased from www.ccf.com.cn from January 29, 2018 to March 4, 2022.

The factors affecting the price of polyester yarn are complex. In order to reduce the prediction bias that may be caused by omission of independent variables, combined with the existing research literature, this paper collects industry data from multiple sources as the independent variables of the prediction model.

The data on daily main contract settlement price of PTA is drawn from Zhengzhou Commodity Exchange. Considering that MEG futures was not listed by Dalian Commodity Exchange before December 10, 2018, the data on daily main contract settlement price of MEG is from two sources, including Dalian Commodity Exchange and Huaxicun Commodity Contracts Exchange. Data on monthly average production load of polyester factory and weekly average operating rate of looms in Jiangsu and Zhejiang provinces are from data services purchased from www.ccf.com.cn. Daily settlement price of Brent crude oil is generated from Sina. The dataset used for the analysis is presented in Table Raw Data in S1 File.

As the direct raw materials for producing polyester yarn, the prices of PTA and MEG reflect the cost of producing polyester yarn. Monthly average production load of polyester factory represents the production capacity of polyester yarn. Weekly average operating rate of looms in Jiangsu and Zhejiang provinces represents the demand market of the downstream industry. Meanwhile, since polyester yarn is a petroleum product, the fluctuation of Brent crude oil price is transmitted through the polyester textile industry chain. It affects the price trend of polyester yarn from multiple dimensions such as raw material cost and market sentiment.

Based on the above analysis, this paper sets the initial model of polyester yarn price forecast as:

$$Y=\beta_{0}+\beta_{1}X_{1}+\beta_{2}X_{2}+\beta_{3}X_{3}+\beta_{4}X_{4}+\beta_{5}X_{5}+\varepsilon$$ (2)

*Y* represents the daily average price of 50D/24F FDY. *X*_1_ is daily main contract settlement price of PTA. *X*_2_ means daily main contract settlement price of MEG. *X*_3_ represents monthly average production load of polyester factory. *X*_4_ is weekly average operating rate of looms in Jiangsu and Zhejiang provinces. *X*_5_ stands for daily settlement price of Brent crude oil.

## 4. Analysis

**Fig 1:**
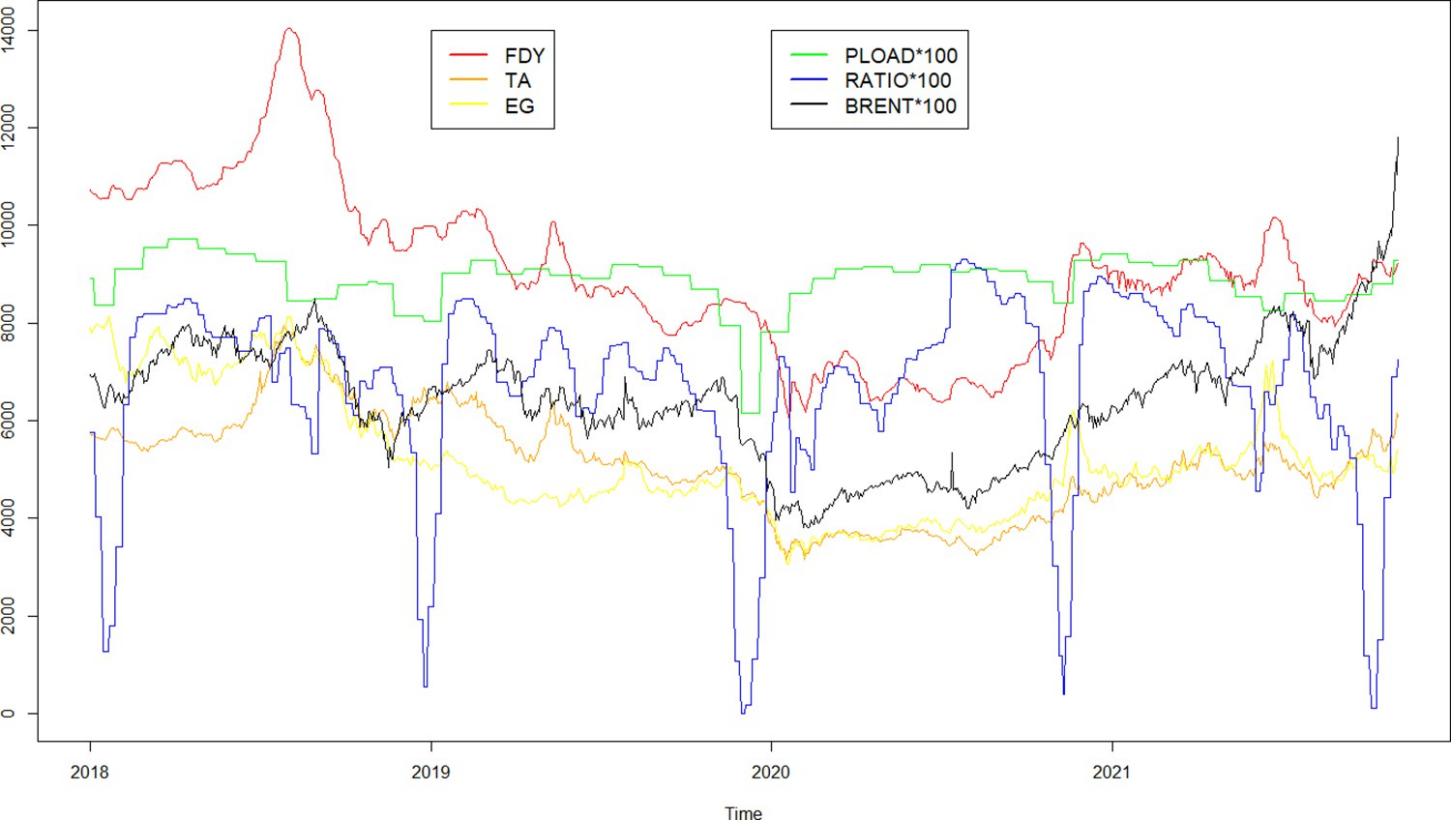
Historical price fluctuations.

Fig 1 describes the fluctuation of each dependent variable and independent variable for January 29, 2018 to March 4, 2022. FDY in the figures represents the daily average price of 50D/24F FDY. TA means daily main contract settlement price of PTA. EG stands for daily main contract settlement price of MEG. PLOAD represents monthly average production load of polyester factory. RATIO is weekly average operating rate of looms in Jiangsu and Zhejiang provinces. BRENT means daily settlement price of Brent crude oil.

Since polyester textile production in China is mainly concentrated in Jiangsu and Zhejiang provinces, RATIO selects the loom operating rates in these two provinces. Additionally, as most polyester textile enterprises suspend operations during the Chinese New Year holiday, some of the time-point values in the RATIO data are close to zero.

### 4.1 Intuitive analysis

**Fig 2:**
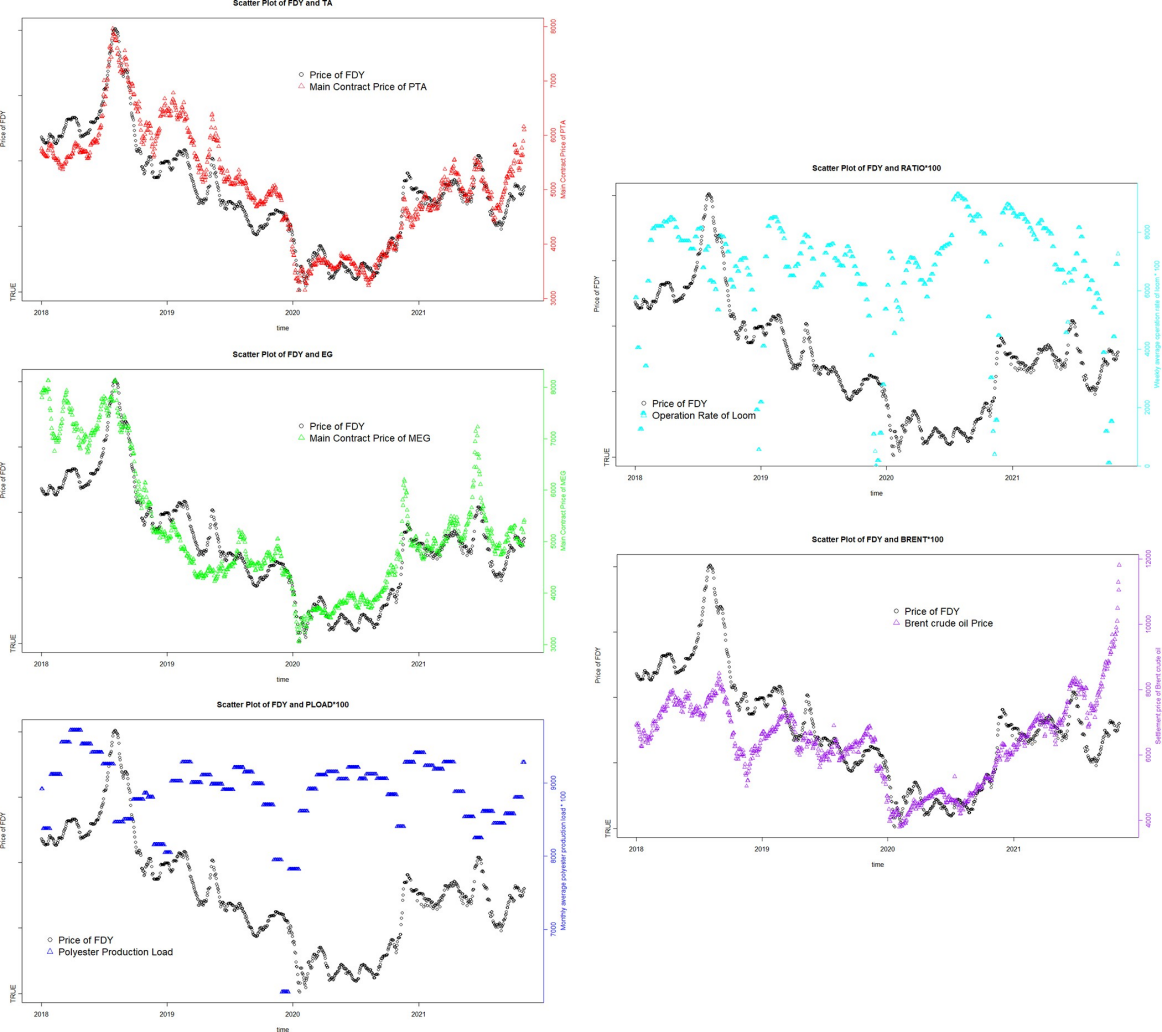
Scatter plot analysis.

Fig 2 demonstrates that the price of polyester yarn has a relatively significant correlation with the prices of PTA, MEG and Brent crude oil. The production load of polyester factory and the operating rate of looms, which represent the upstream and downstream supply and demand, have some degree of impact on the fluctuation of polyester yarn prices.

Since magnitudes of different variables differs greatly, this paper standardizes data for all variables to make all variables having same magnitude, using:

$$sX=\frac{X-mean(X)}{sigma\;of\;X}$$ (3)

**Fig 3:**
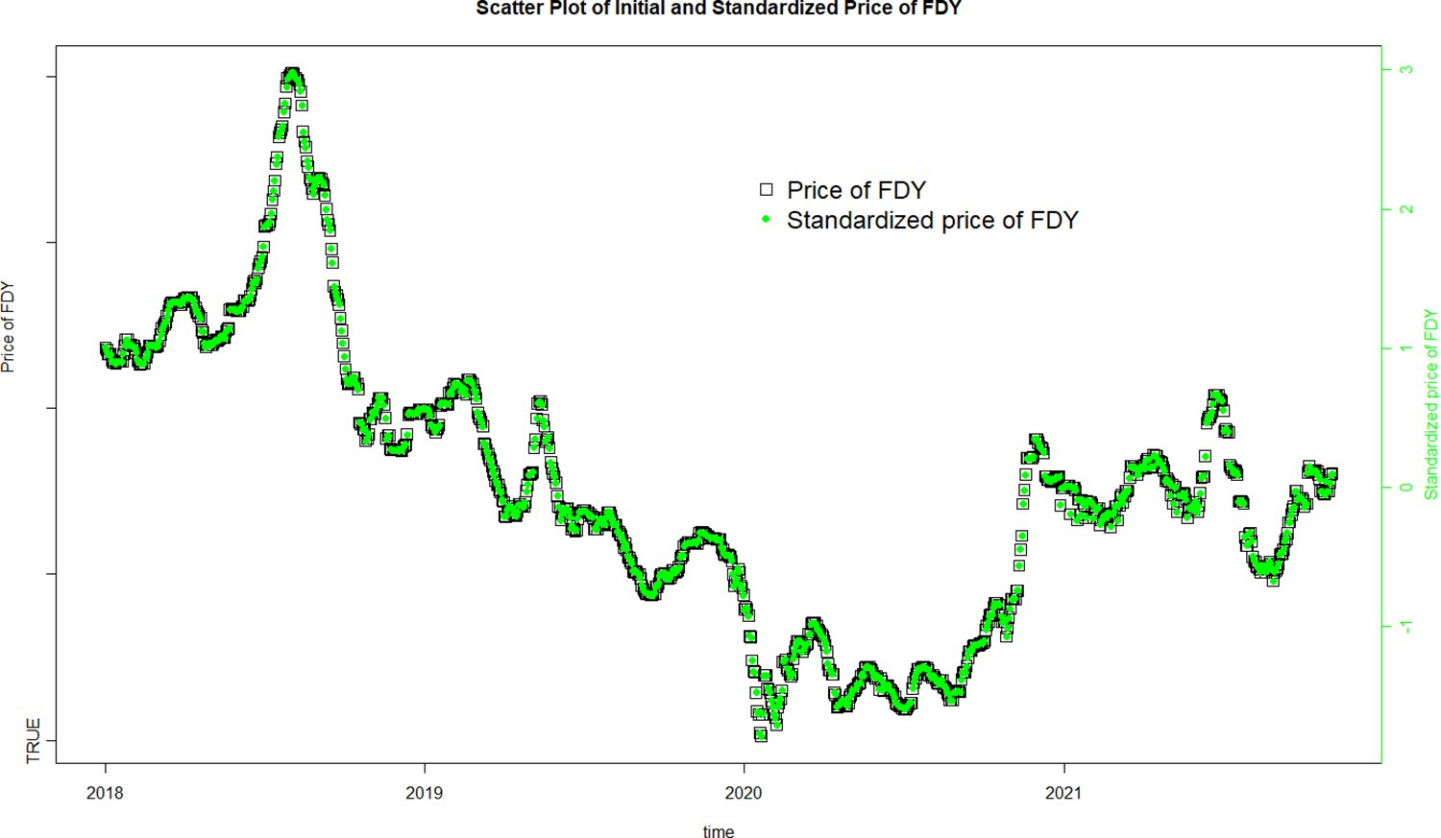
Standardized data inspection.

Fig 3 demonstrates that the standardized data has same trend with initial data. Thus, it is reliable to use standardized data in the prediction model.

### 4.2 Linear relationship test

**Fig 4:**
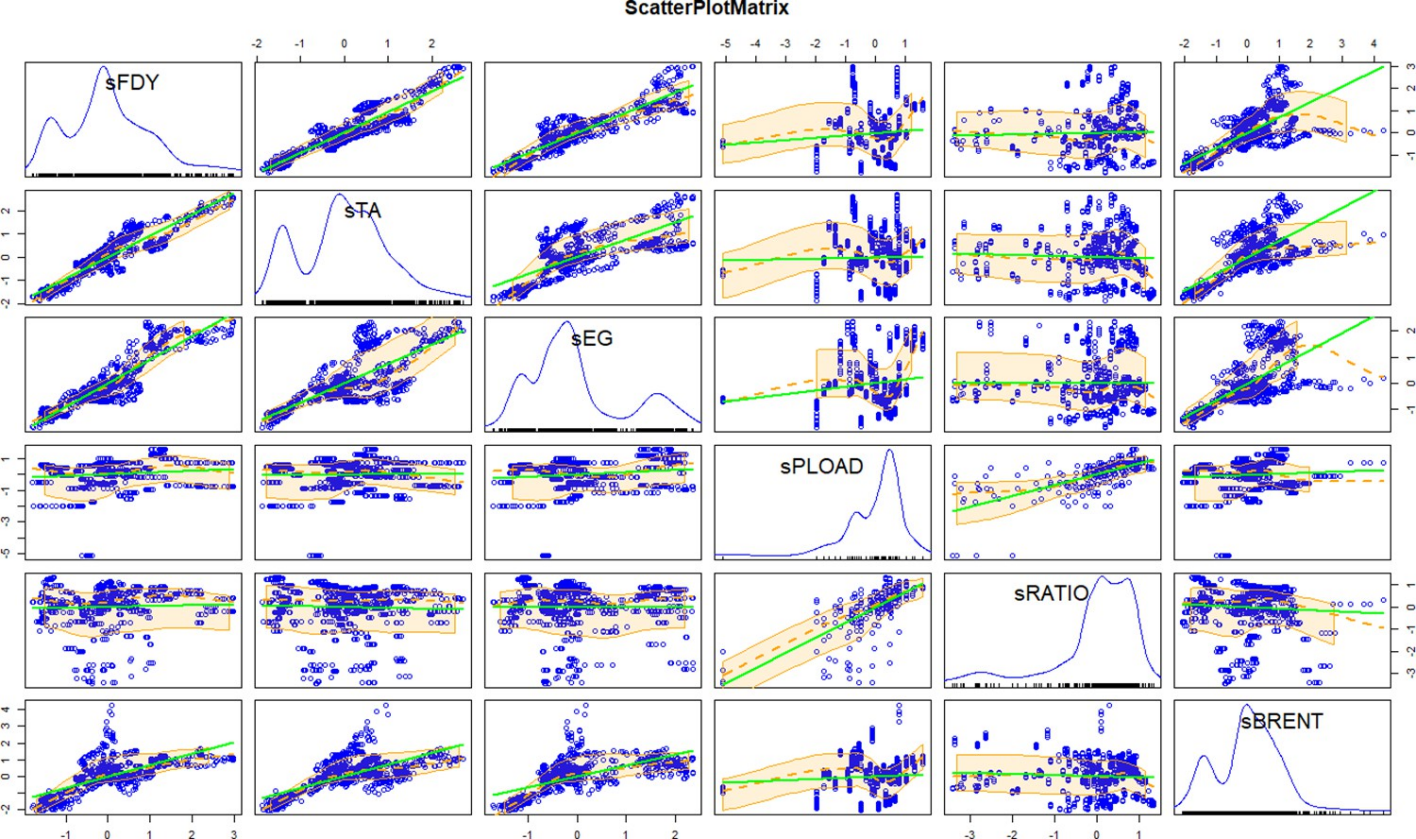
Linear relationship test.

Fig 4 shows that there are significant linear relationships among polyester yarn price and PTA price, MEG price, crude oil price. There are some degree of linear relationship between polyester yarn price and the production load of polyester factory, or the operating rate of looms. This requires further testing.

### 4.3 Stationary test

This paper uses Phillips-Perron Unit Root Test to test whether dependent variable and independent variables are stationary.

Null Hypothesis: The time series data has a unit root and is non-stationary.

Alternative Hypothesis: The time series data is stationary and does not have a unit root.

Table 1 indicates that most dependent variable and independent variables are non- stationary. Thus, it is necessary to have variables cointegrated and residual stationary.

**Table 1:** Phillips-Perron unit root test result.

Variable	Definition	p-value
sFDY.ts	Standardized daily average price of 50D/24F FDY	0.8834
sTA.ts	Standardized daily main contract settlement price of PTA	0.9627
sEG.ts	Standardized daily main contract settlement price of MEG	0.8023
sPLOAD.ts	Standardized monthly average production load of polyester factory	0.04392
sRATIO.ts	Standardized weekly average operating rate of looms in Jiangsu and Zhejiang provinces	0.01
sBRENT.ts	Standardized daily settlement price of Brent crude oil	0.99

### 4.4 Cointegration test

This paper uses Johansen-Procedure Test to test whether dependent variable and independent variables are cointegrated.

Null Hypothesis: There is 0 cointegrated vector.

Alternative Hypothesis: There exists at least one cointegration relationship in the system.

Table 2 shows that it is valid to reject null hypothesis at 1% significant level since 120.34 is greater than 104.20. Thus, dependent variable and independent variables are cointegrated.

**Table 2:** Johansen-Procedure test result.

	α	test	0.10	0.05	0.01
r = 0	0.01	120.34	85.18	90.39	104.20

### 4.5 Regression analysis

The empirical model is based on previous analysis. Let sFDY represents standardized daily average price of 50D/24F FDY. sTA represents standardized daily main contract settlement price of PTA. sEG represents standardized daily main contract settlement price of MEG. sPLOAD represents standardized monthly average production load of polyester factory. sRATIO represents standardized weekly average operating rate of looms in Jiangsu and Zhejiang provinces. sBRENT represents standardized daily settlement price of Brent crude oil.


$${sFDY}_{t}=\beta_{0}+\beta_{1}{sTA}_{t}+\beta_{2}{sEG}_{t}+\beta_{3}{sPLOAD}_{t}+\beta_{4}{sRATIO}_{t}+\beta_{5}{sBRENT}_{t}+\varepsilon_{t}$$ (4)

Since the linear relationship between polyester yarn price and the production load of polyester factory, or the operating rate of looms, is not very significant, this paper sets up another model leaving out these two independent variables and compares results from these two models.


$${sFDY}_{t}=\beta_{0}+\beta_{1}{sTA}_{t}+\beta_{2}{sEG}_{t}+\beta_{3}{sBRENT}_{t}+\varepsilon_{t}$$ (5)

Since p-value is less than 0.01, Table 3 indicates that, in addition to the price of PTA, MEG and Brent crude oil, the production load of polyester factory and the operating rate of looms also have significant impact on the price of polyester yarn at the 1% significance level.

Null Hypothesis: The regression coefficient is equal to zero and is not statistically significant.

Alternative Hypothesis: The regression coefficient is not equal to zero and is statistically significant.

**Table 3:** Regression result (p-value in parentheses).

	Definition	(4)	(5)
(Intercept)	Regression constant	-2.909e-16 (1)	-2.420e-16 (1)
sTA	Standardized daily main contract settlement price of PTA	5.792e-01 (< 2e-16)	5.819e-01 (< 2e-16)
sEG	Standardized daily main contract settlement price of MEG	4.980e-01 (< 2e-16)	4.977e-01 (< 2e-16)
sPLOAD	Standardized monthly average production load of polyester factory	-3.000e-02 (0.001392)	N.A.
sRATIO	Standardized weekly average operating rate of looms in Jiangsu and Zhejiang provinces	8.256e-02 (< 2e-16)	N.A.
sBRENT	Standardized daily settlement price of Brent crude oil	-3.538e-02 (0.000368)	-4.496e-02 1.4e-05

In addition, AIC (Akaike Information Criterion) Test result (Table 4) also shows it is necessary to consider these two independent variables into model. Thus, this model uses Model (4) as regression function.

Null Hypothesis: All candidate models possess equal explanatory power and predictive performance.

Alternative Hypothesis: Among the models being compared, at least one model outperforms the others in terms of explaining the data or predicting future observations.

**Table 4:** AIC test result.

	(4)	(5)
AIC	-298.9517	-210.3863

### 4.6 Stationary residual test

This paper uses Phillips-Perron Unit Root Test to test the stationarity of residual. The Phillips-Perron Unit Root Test result is:

Dickey-Fuller = -4.9743, Truncation lag parameter = 7, p-value = 0.01.

Null Hypothesis: The time series data has a unit root and is non-stationary.

Alternative Hypothesis: The time series data is stationary and does not have a unit root.

Since p-value is less than 0.05, so, it is reliable to reject the null hypothesis at 95% confidence interval. Thus, residual is stationary.

Because dependent variable and independent variables are cointegrated and the residual is stationary, the result from regression model (4) is reliable.

### 4.7 Multicollinearity test

This paper uses VIF Test to test whether there is multicollinearity in the regression model.

Table 5 proves that there is no multicollinearity in the regression model since all test results are less than 10.

Null Hypothesis: There is no multicollinearity among the independent variables.

Alternative Hypothesis: There is multicollinearity among the independent variables.

**Table 5:** VIF test result.

sTA	sEG	sPLOAD	sRATIO	sBRENT
6.164446	6.164446	6.164446	6.164446	6.164446

### 4.8 Model fitness test

**Fig 5:**
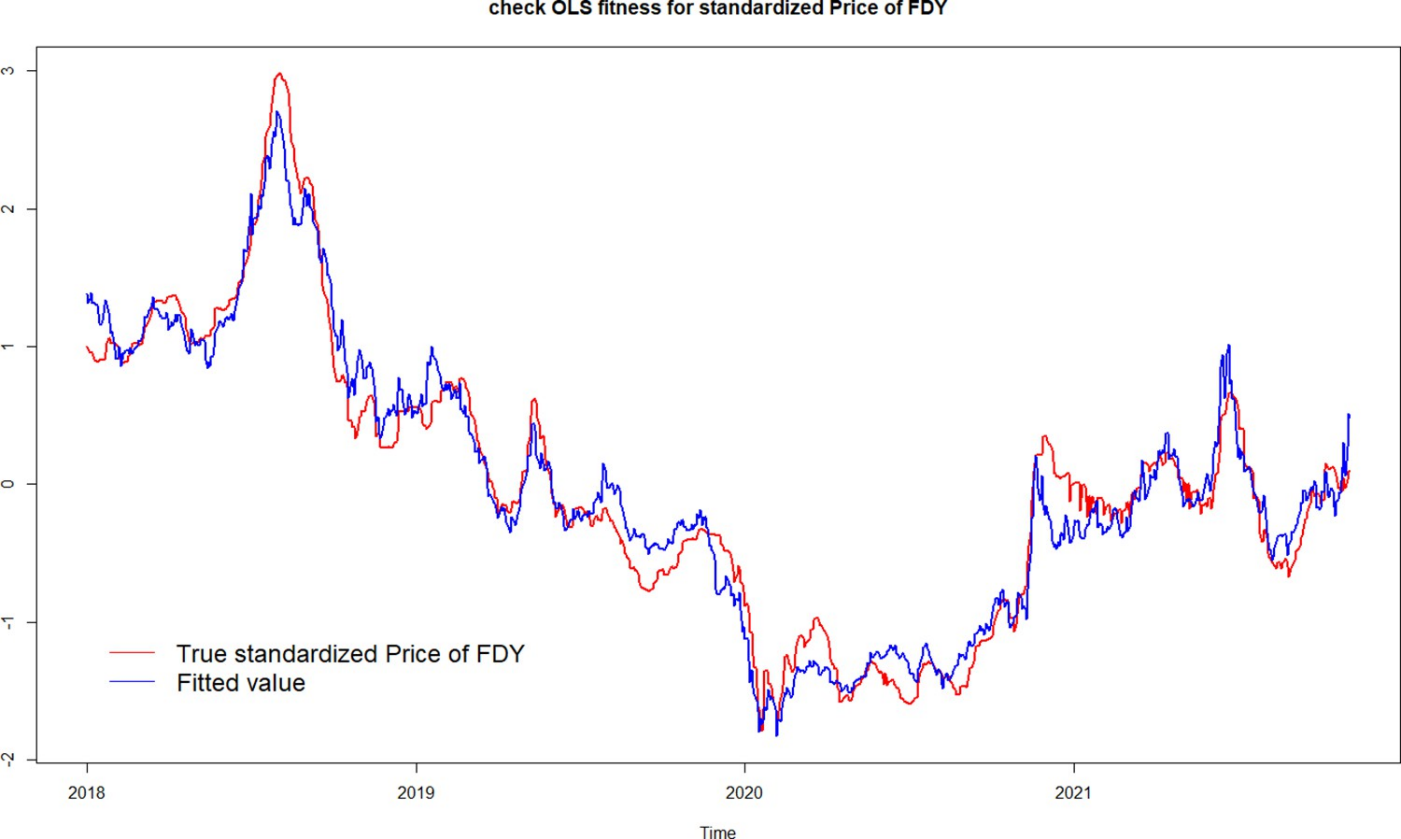
Regression model fitting results.

In Fig 5, the red line represents the actual historical values, while the blue line represents the fitted values obtained using the regression model in this study. The figure visually demonstrates that the overall trend of the blue fitted values is consistent with the red actual values, with similar time points for both upward and downward movements, and a relatively small numerical difference. Therefore, through the fitting test of historical actual values, it can be concluded that the regression model used in this study fits well.

### 4.9 Forecast

This paper uses the Holt-Winters model to predict the value of each independent variable in the next 30 days.

**Fig 6:**
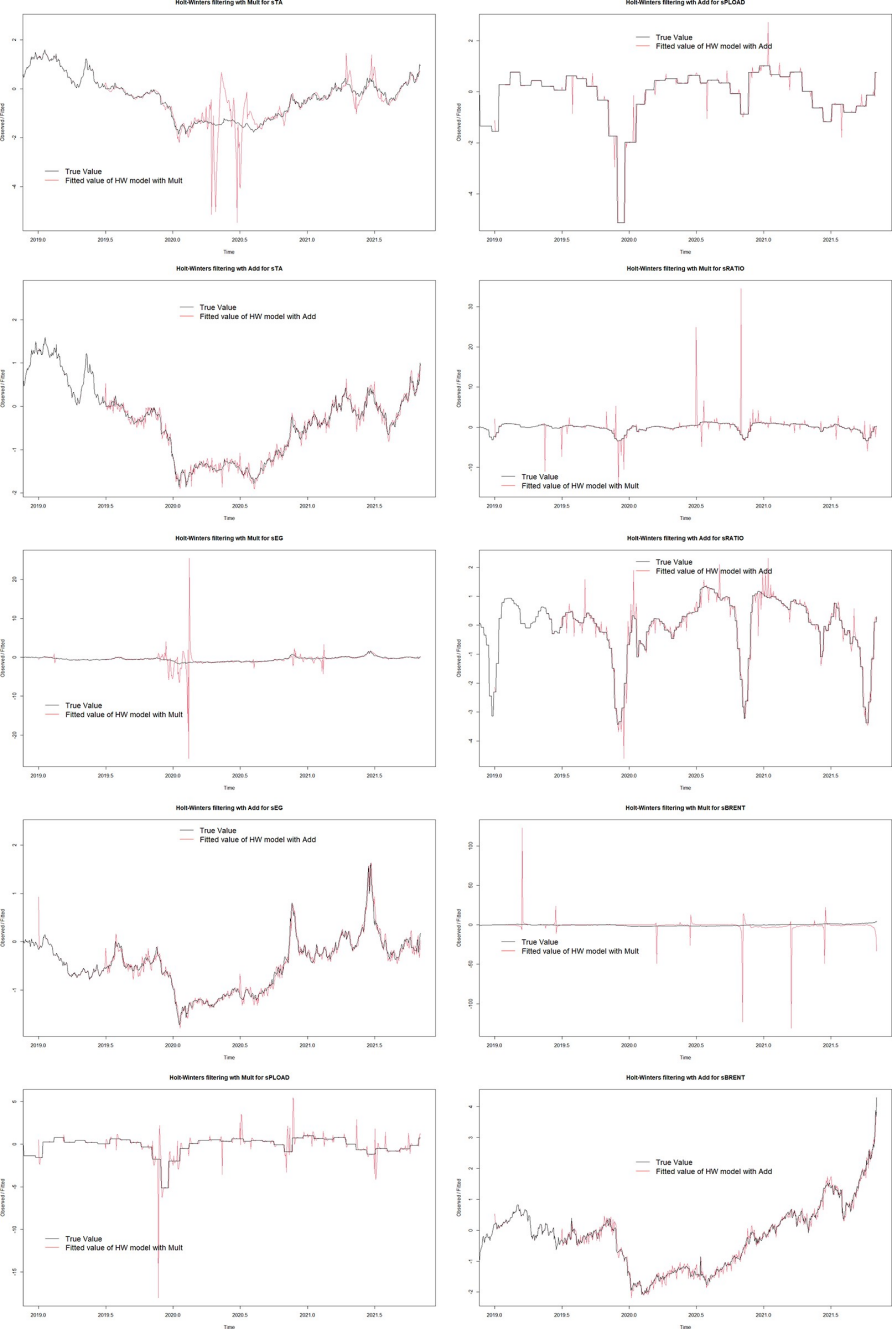
Holt-Winters model fit test.

Fig 6 indicates that the fit of the Cumulative Triple Exponential Smoothing with Additive Model (as shown in Fig 6B, 6D, 6F, 6H, and 6J) is better than that of the Cumulative Triple Exponential Smoothing with Multiplicative Model (as shown in Fig 6A, 6C, 6E, 6G, and 6I). Therefore, the Cumulative Triple Exponential Smoothing with Additive Model is selected.

**Fig 7:**
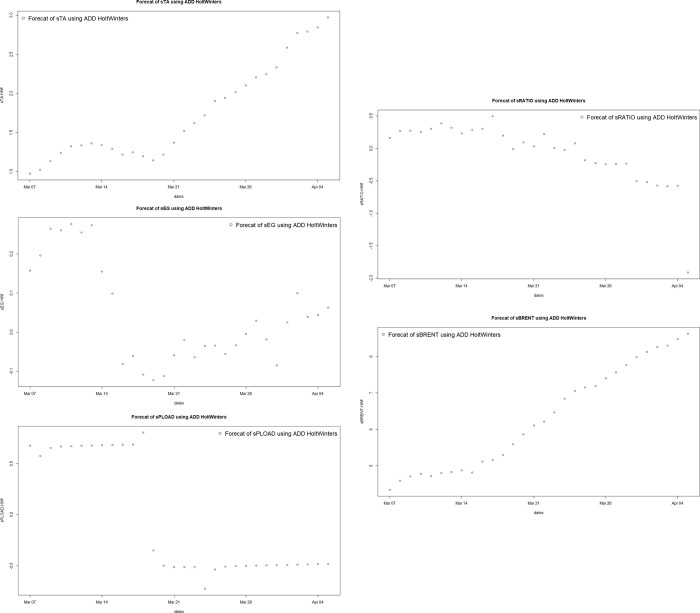
Holt-Winters model predictions.

Fig 7 displays the prediction results of the values of independent variables for the next 30 days using the Holt-Winters model, specifically the Cumulative Triple Exponential Smoothing with Additive Model. Fig 7(A)–7(E) respectively represent the predicted values of standardized daily main contract settlement price of PTA, standardized daily main contract settlement price of MEG, standardized monthly average production load of polyester factory, standardized weekly average operating rate of looms in Jiangsu and Zhejiang provinces, and standardized daily settlement price of Brent crude oil for the next 30 days.

In this paper, model (4) is used to predict the standardized daily average price of 50D/24F FDY in the next 30 days, with the predicted values of the independent variables for the future 30 days set as prediction results obtained from the Holt-Winters model as shown in Fig 7.

**Fig 8:**
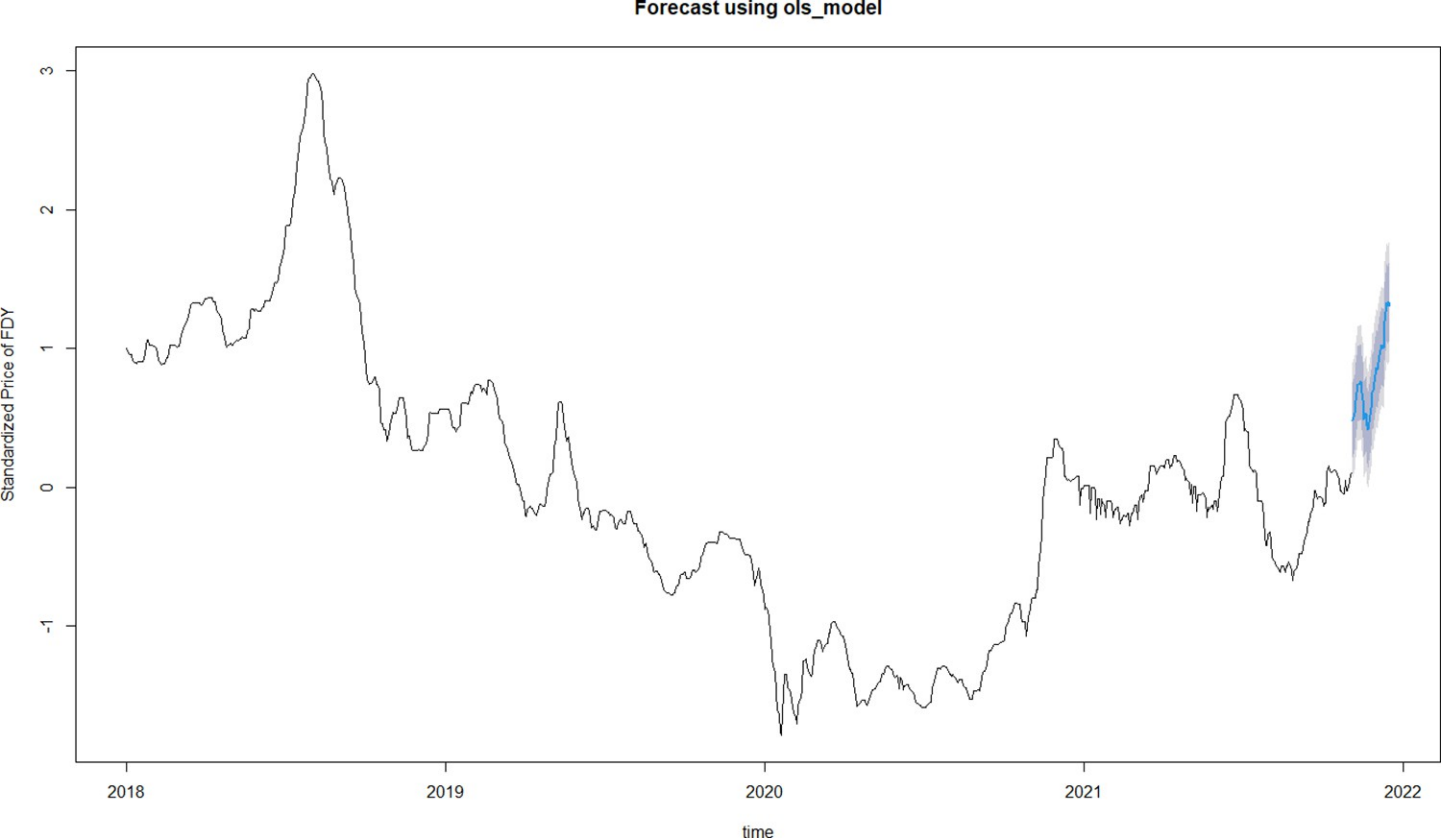
Forecast trend of standardized price of FDY.

Fig 8 and Table 6 describe the prediction results.

**Table 6:** Forecast value of standardized price of FDY.

Data	Point Forecast: Standardized Price of FDY	Point Forecast: Price of FDY
2022-03-07	0.4835392	9859.071
2022-03-08	0.5344802	9944.468
2022-03-09	0.6244313	10095.262
2022-03-10	0.6805273	10189.301
2022-03-11	0.7425837	10293.332
2022-03-14	0.7430089	10294.044
2022-03-15	0.7598855	10322.336
2022-03-16	0.6800135	10188.439
2022-03-17	0.6311120	10106.461
2022-03-18	0.4884443	9867.294

**Fig 9:**
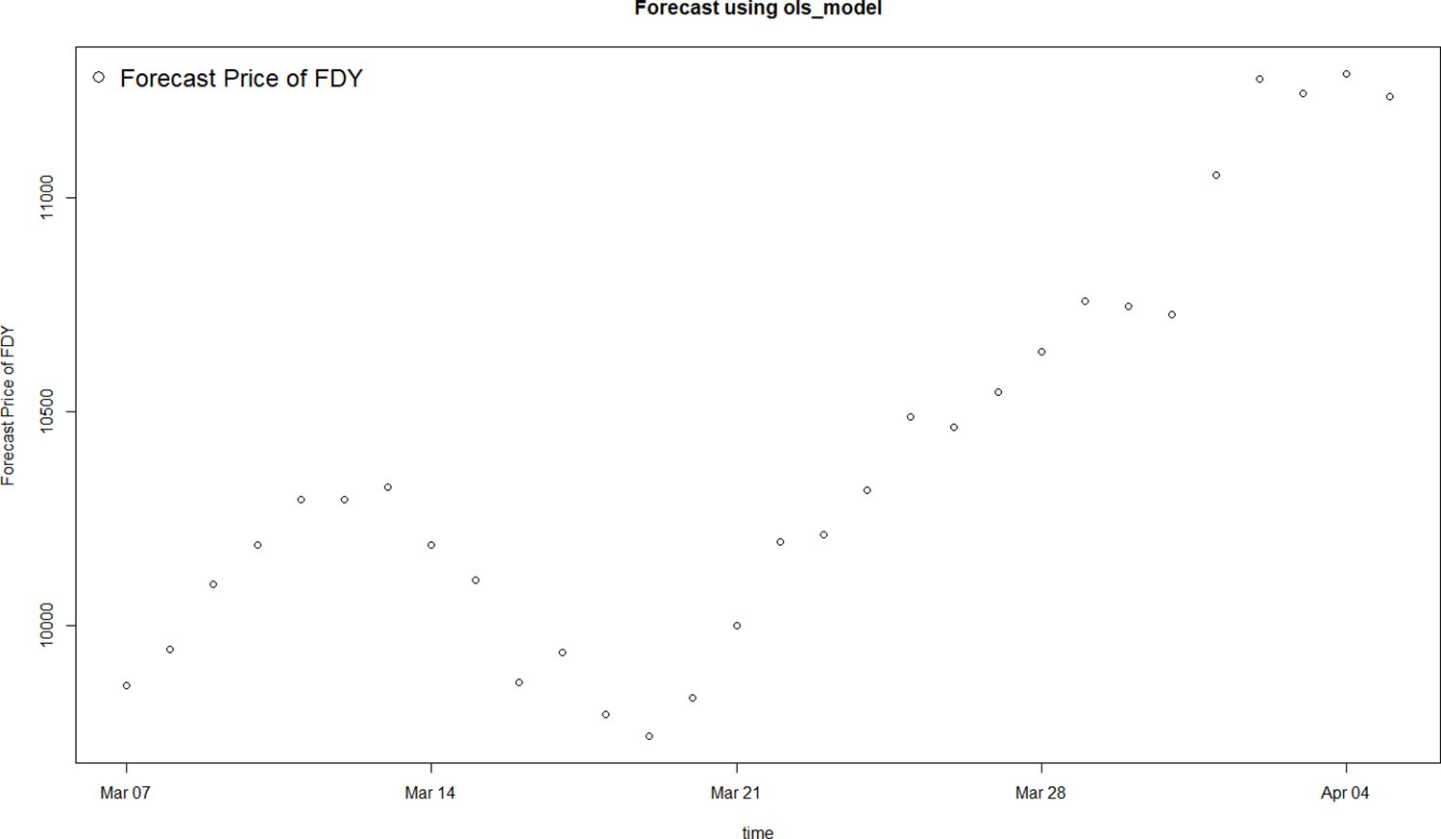
Forecast price of 50D/24F FDY.

Convert the standardized value into the absolute value of daily average price of 50D/24F FDY. Fig 9 shows the forecast results of polyester yarn prices in the next 30 days.

**Fig 10:**
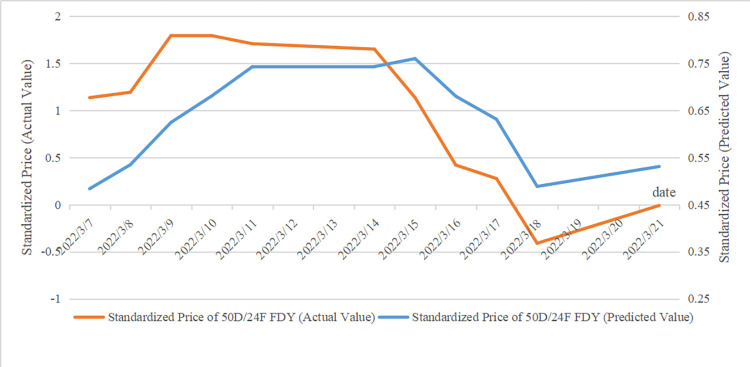
Comparison of the trends between actual and predicted values.

Since the model is used to predict price fluctuations over a period of time after a certain date, unexpected events during that period can easily lead to consistent errors in absolute values, while the impact on the trend is minor. Therefore, the focus of the model is on capturing the general direction of price movements rather than the precise numerical values. Table 7 presents the predicted and actual values after standardization, while Fig 10 compares the fluctuation trends of the predicted and actual values. As shown in Fig 10, the predicted price shows a trend of first rising, then stabilizing for about three working days, and facing a decline afterwards. After that, an upward trend is expected. It is evident that the overall trend of price fluctuations is consistent between actual and predicted value.

**Table 7:** Comparison between actual values and predicted values.

Data	Standardized Price of FDY (Actual Value)	Standardized Price of FDY (Predicted Value)
2022-03-07	1.13494018	0.4835392
2022-03-08	1.19221218	0.5344802
2022-03-09	1.79356821	0.6244313
2022-03-10	1.79356821	0.6805273
2022-03-11	1.70766021	0.7425837
2022-03-14	1.6503882	0.7430089
2022-03-15	1.13494018	0.7598855
2022-03-16	0.41904015	0.6800135
2022-03-17	0.27586015	0.631112
2022-03-18	-0.41140388	0.4884443
2022-03-21	-0.01049987	0.5307096

Therefore, textile enterprises can view the short-term rise in raw material prices more rationally, wait for prices to fall, and optimize the timing of raw material procurement. For traders holding polyester yarn inventory, the price rising period might be a good opportunity to sell. It is advisable for traders to consider appropriate promotions to reduce inventory, and then restock when prices fall.

## 5. Conclusion

In conclusion, the price of polyester yarn is significantly related to PTA price, MEG price, production load of polyester factory, operating rate of looms, and Brent crude oil price.

This conclusion is basically consistent with the theoretical analysis results. As the raw materials of polyester yarn, the increase of PTA price and MEG price will push up the price of polyester yarn. Production load of polyester factory represents the production capacity of polyester yarn. Under the condition that demand remains unchanged, higher production capacity will lead to a decrease in the price of polyester yarn. Operating rate of looms represents the demand market. Under the condition of constant supply, higher demand will lead to an increase in the price of polyester yarn.

Mastering this model is helpful for relevant enterprises to avoid price risk and reduce production costs. However, in the midst of market volatility, quantitative model analysis may intensify panic, which can easily trigger speculation.

In addition, when employing quantitative models, special emphasis should be placed on data ethics principles. The rights of data producers regarding the storage, deletion, use, and dissemination of data should be fully respected. In this paper, manufacturing enterprises, as producers of data, are the primary community that the model should serve.

## Supporting information

S1 File (CSV)
